# Evaluation of plant growth promotion properties and induction of antioxidative defense mechanism by tea rhizobacteria of Darjeeling, India

**DOI:** 10.1038/s41598-020-72439-z

**Published:** 2020-09-23

**Authors:** Chandrima Bhattacharyya, Srimoyee Banerjee, Udita Acharya, Aroni Mitra, Ivy Mallick, Anwesha Haldar, Shyamalina Haldar, Anupama Ghosh, Abhrajyoti Ghosh

**Affiliations:** 1 Department of Biochemistry, Bose Institute, Centenary Campus, P1/12, C.I.T. Road, Scheme VIIM, Kolkata, 700054 India; 2 Division of Plant Biology, Bose Institute, Centenary Campus, P1/12, C.I.T. Road, Scheme VIIM, Kolkata, 700054 India; 3 Department of Geography, University of Calcutta, 35 Ballygunge Circular Road, Kolkata, 700019 India; 4 Department of Biochemistry, Asutosh College, University of Calcutta, Kolkata, 700026 India

**Keywords:** Enzymes, Agroecology, Applied microbiology, Bacteria, Symbiosis, Soil microbiology, Microbiology, Soil microbiology

## Abstract

A total of 120 rhizobacteria were isolated from seven different tea estates of Darjeeling, West Bengal, India. Based on a functional screening of in vitro plant growth-promoting (PGP) activities, thirty potential rhizobacterial isolates were selected for in-planta evaluation of PGP activities in rice and maize crops. All the thirty rhizobacterial isolates were identified using partial 16S rRNA gene sequencing. Out of thirty rhizobacteria, sixteen (53.3%) isolates belong to genus Bacillus, five (16.6%) represent genus Staphylococcus, three (10%) represent genus Ochrobactrum, and one (3.3%) isolate each belongs to genera Pseudomonas, Lysinibacillus, Micrococcus, Leifsonia, Exiguobacterium, and Arthrobacter. Treatment of rice and maize seedlings with these thirty rhizobacterial isolates resulted in growth promotion. Besides, rhizobacterial treatment in rice triggered enzymatic [ascorbate peroxidase (APX), catalase (CAT), chitinase, and phenylalanine ammonia-lyase (PAL)], and non-enzymatic [proline and polyphenolics] antioxidative defense reactions indicating their possible role in the reduction of reactive oxygen species (ROS) burden and thereby priming of plants towards stress mitigation. To understand such a possibility, we tested the effect of rhizobacterial consortia on biotic stress tolerance of rice against necrotrophic fungi, *Rhizoctonia solani* AG1-IA. Our results indicated that the pretreatment with rhizobacterial consortia increased resistance of the rice plants towards the common foliar pathogen like *R*. *solani* AG1-IA. This study supports the idea of the application of plant growth-promoting rhizobacterial consortia in sustainable crop practice through the management of biotic stress under field conditions.

## Introduction

A diverse population of soil bacteria usually inhabits plant rhizospheres. A subset of these is beneficial to the plants in terms of promoting their growth. These bacteria, therefore, are very commonly referred to as plant growth-promoting rhizobacteria (PGPR) and serve as potential environment-friendly substitutes of chemical fertilizers^1,2^. Phytostimulating rhizobacteria, in general, do not show host specificity and can exhibit their growth-promoting features when associated with a broad range of hosts^3^. This makes them very suitable as biofertilizers as their application can be extended to multiple plants that might not serve as their natural hosts. In the case of biocontrol, for endophytic and mycorrhiza helper PGPR, however, genotype-dependent specificity in PGPR- plant cooperation has been reported^3,4^. PGPR contributes to the general well-being of the associated plants either by direct or indirect manner^1^. In the direct mechanism, PGPR stimulates plant growth by fixing atmospheric nitrogen, solubilizing soil insoluble phosphates and potassium, making iron available for the host plant, and finally, by producing different phytohormones to support plant growth^1^. The indirect mechanism of PGPR involves the protection of plants from biotic and abiotic stresses. The major indirect mechanisms adopted by PGPR are hydrolytic enzyme production, exo-polysaccharide production, bioremediation of heavy metals, and stimulation of induced systemic resistance (ISR)^1^. In plants, PGPR activates the immune response by stimulating an induced systemic resistance (ISR) through strengthening the physical and biochemical responses of the plant cell towards environmental stresses^5,6^. Association of PGPR with host plants has often been found to enhance the biosynthesis of defense-related molecules in the later. The elevated levels of the defense proteins thus provide the host plant a better chance of survival under stress conditions^5,6^. Both biotic, as well as abiotic stresses, impose a number of different physiological changes in plant cells, including the generation of reactive oxygen species (ROS)^7^. Accumulation of a high concentration of ROS in plant cells leads to oxidative damage and results in the disruption of cellular homeostasis^7^. Plant cells are equipped with sophisticated antioxidative mechanisms involving antioxidative defense enzymes like Ascorbate peroxidase (APX), Catalase (CAT), peroxidase (PO), superoxide dismutase (SOD), glutathione reductase, glutathione S-transferase and guaiacol peroxidase^8^. These enzymes are involved in scavenging and transforming ROS into non-toxic end products and thereby protect cells from oxidative damage^8^. Besides, plant cells also produce various antioxidant molecules such as carotenoids and phenylpropanoids to overcome oxidative damage^9^. PGPR mediated ISR primes host plants towards resisting pathogen invasion through the production of defense-related antioxidative enzymes and molecules^5^.

Tea (*Camellia sinensis* (L.) O. Kuntze.) is an economically important perennial crop plant mostly cultivated in the North-Eastern part in India. Extensive use of agrochemicals to meet the global requirement of tea resulted in an alteration of the microbial community associated with the tea plants^10,11^. Interestingly, little is known regarding the tea rhizosphere microbiome of Indian tea. Several culture-dependent analyses have shown that the tea rhizosphere is constituted of a variety of metabolically versatile PGPR that has the potential to be used as biofertilizer^12–16^. Moreover, a few of these rhizobacteria were also found to act as biocontrol agents^12,13,17,18^. However, a lack of systematic analysis combining different rhizobacterial isolates rendered only a little progress in the development of consortia-based biofertilizer formulations.

In the present study, we report isolation, characterization, and plant growth-promoting (PGP) activities of 30 rhizobacteria isolated from the rhizospheric soils of seven tea estates in Darjeeling, West Bengal. All the rhizobacterial isolates were tested for their PGP activities in two major crop plants, rice, and maize. After tea, rice and maize together cover the majority of the cultivable crop area in Darjeeling region^19^ (Source: https://darjeeling.gov.in/agriculture.html). However, their yields are significantly affected due to several factors, including local agro-climatic conditions and conventional agricultural practices^19,20^. The soil in this region is mostly acidic, leading to low availability of essential base cations and phosphate^21,22^. Moreover, the use of local low yielding seed varieties contributes towards the reduced total production of the food grains. Decreasing productivity of these crops raises further concern about food security and the social livelihood of the local farming population. Application of PGPR might come to a great rescue under such a circumstance. PGPR have well-established properties of restoring the bioavailability of nutrients in addition to promoting crop growth. Besides, we also evaluated the effect of rhizobacterial treatment on the production of antioxidative defense enzymes and molecules in rice plants. Furthermore, we took a consortia based approach to evaluate the effect of rhizobacterial treatment on rice plants in inducing resistance towards infection with necrotrophic fungi *Rhizoctonia solani* AG1-IA leading to sheath blight disease.

## Materials and methods

### Geography of the terrain of the study sites

The Darjeeling District is mostly set over the Himalayan hill region in the north, and Terai plains in the south with the local relief varying from 100 m up to 3,636 m in Sandakphu peak intersected largely by tributary streams of the Teesta, Mahanadi and Jaldhaka rivers. The amount of precipitation influences the slope stability and vegetation of the region. The southern slopes receive 4,000 to 5,000 mm rainfall annually while the northern leeward slopes 2,000–2,500 mm, concentrating over 218 rainy days annually. Temperature isolines have a wider range both seasonally as well as over the district. The vegetation sharply progresses from the moist tropical deciduous forests from 300–1,000 m to evergreen montane forests to 3,000 m, and temperate forests above 3,000 m. Geologically, the district comprises of relatively recent rock structures. The sub-Himalayas are made up of Siwalik systems composed of mudstones, sandstones, shale, and conglomerates, followed by lower Godowana systems or Damuda series that comprise the outer region. In contrast, the Daling series group of crystalline rocks composed of hard-grained sandstone chlorite shales, phyllites and schists associated with quartzites form towards the inner Himalayas^23,24^. Of the 2,417 km^2^ area of the Darjeeling hills, 18% is covered under the tea plantations. The tea gardens across the district are mostly situated on steep slopes where well-drained podzolic soils are in abundance.

### Sampling of tea rhizosphere soil

**Figure 1:**
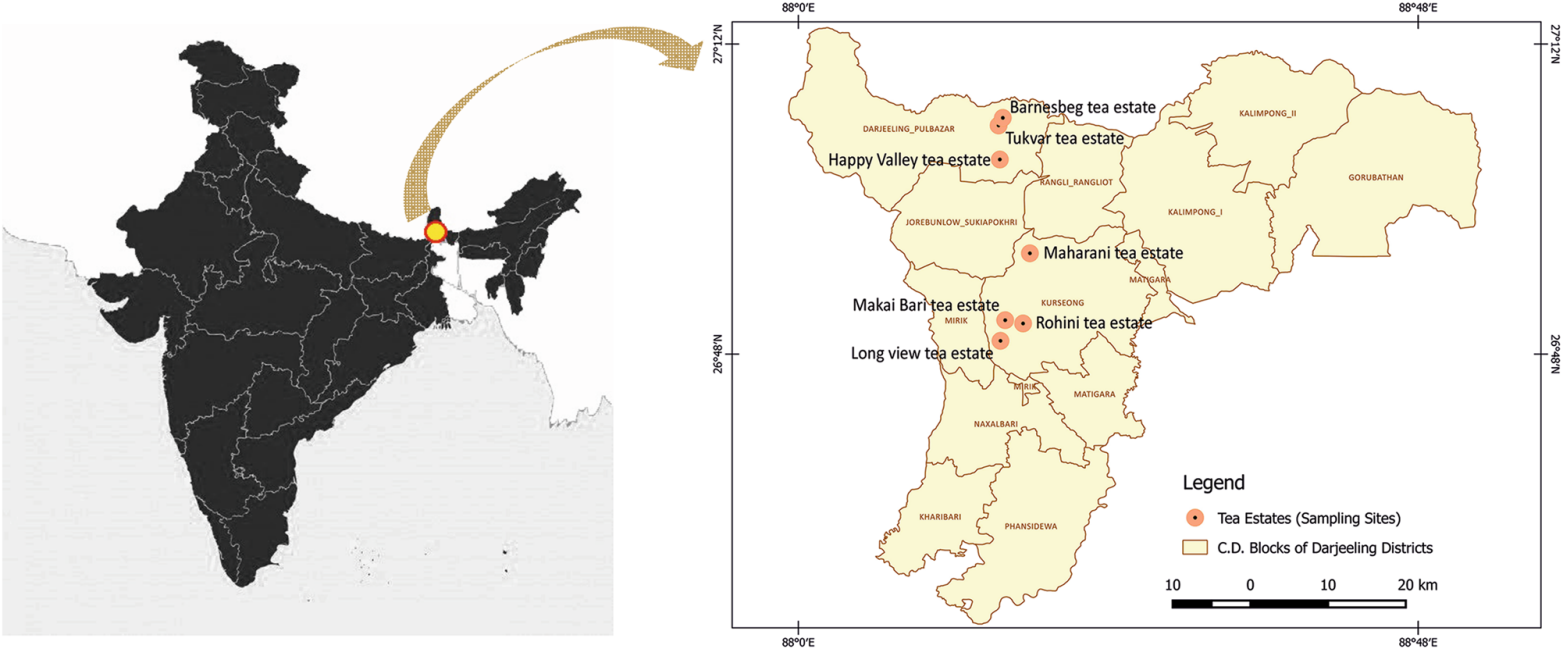
The geographical location of the sampling tea estates in the Darjeeling district of West Bengal, India. Coordinates of the sampling tea estates and description of the stations are presented in Table 1.

The rhizospheric soil samples of tea plants were collected in May 2016 from seven different tea estates of Darjeeling, India. Detailed site characteristics are provided in Table 1 and Fig. 1. Soil samples were collected from the rhizosphere area at 20 ± 2 cm depth. The area of the tea estates was divided into three zones, from each zone samples were collected from four randomly selected healthy tea plants containing roots and root-adhered soil. Finally, all four rhizosphere soil samples were pooled, resulting in a total of three composite rhizosphere soil samples from each tea estate. All the samples were immediately stored at 4 °C and subsequently either directly used for chemical analysis and microbiological analysis or stored at − 80 °C for molecular biological studies.

**Table 1:** The location, soil type and number of total bacteria isolated from tea rhizosphere soils collected from the sampling sites in the tea estate of Darjeeling.

Sampling sites (tea estates)	Coordinates (latitude and longitude)	Soil type (USDA)	Soil pH	Total soil bacterial count (CFU g^−1^ range)	Number of isolates (number used for downstream analysis)
Tukvar tea estate	27° 5′42.01ʺ N; 88°15′30.24ʺ E	Sandy loam, Silty	4.7	3 × 10^4^ − 3 × 10^6^	15 (3)
Barnesbeg tea estate	27° 6′17.23ʺ N; 88°15′49.92ʺ E	Sandy loam	4.6	5 × 10^4^ − 6 × 10^6^	17 (5)
Happy Valley Tea estate	27° 3′3.81ʺ N; 88°15′36.12ʺ E	Sandy loam, Silty	4.2	2 × 10^4^ − 3 × 10^6^	13 (3)
Maharani tea estate	26°55′48.36ʺ N; 88°17′55.66ʺ E	Sandy loam and clay	4.6	5 × 10^4^ − 6 × 10^6^	16 (6)
Rohini Tea estate	26°50′22.42ʺ N; 88°17′23.98ʺ E	Sandy loam and clay	4.5	6 × 10^4^ − 7 × 10^6^	17 (4)
Makai Bari Tea estate	26°50′37.67ʺ N; 88°16′0.85ʺ E	Sandy loam	5.0	1 × 10^5^ − 2 × 10^7^	22 (5)
Long view tea estate	26°49′1.75ʺ N; 88°15′39.05ʺ E	Sandy loam	5.2	2 × 10^5^ − 2 × 10^7^	20 (4)

### Isolation of tea rhizobacteria

The tea rhizobacterial strains were isolated using enrichment media following methods described previously^25^. Further functional screening of the isolated rhizobacteria was performed in Pikovskaya Agar (PA), Burks Agar (BA), Jensen’s Agar (JA), *Azotobacter* Agar (AA) (HiMedia Laboratories, India) to screen their ability to show in vitro plant-growth promotion activities. Upon inoculation, the agar plates were incubated either at 30 °C or 37 °C for 24–48 h. The rhizobacterial colonies appeared on the plates were sub-cultured and store at − 80 °C in minimal medium containing 20% glycerol. The morphology and gram-characteristics of the selected rhizobacteria were ascertained using a light microscope (40X, Euromex Oxion trinocular Microscope, Netherlands).

### Molecular identification of the isolates

To identify rhizobacterial isolates, partial amplification of the 16S rRNA gene was carried out using universal primer pair 27F (5′-AGAGTTTGATCMTGGCTCAG-3′) and 1492R (5′-TACGGYTACCTTGTTACGACTT-3′) and genomic DNA as template following protocols described previously^26,27^. The amplified partial rRNA gene was sequenced in ABI 3500 XL Genetic Analyzer with primers 27F, 515F, or 1492R, as described previously^28^.

The evolutionary history of rhizobacterial isolates was inferred using Neighbor-Joining method^29^. The percentages of replicate trees in which the associated taxa clustered together in the bootstrap test (1,000 replicates) are shown next to the branches^30^. The evolutionary distances were computed using the Maximum Composite Likelihood method^31^ and are in the units of the number of base substitutions per site. Evolutionary analyses were conducted in MEGA X^32^. 16S rRNA gene sequence of *Sulfolobus solfataricus* P2 was used to assign an out-group species.

### Phosphate solubilization

For screening of phosphate solubilizing activity of the rhizobacterial isolates, Pikovskaya’s agar plates were used with certain modifications^33^. The reaction was considered positive when a clear halo surrounding the spot inoculated bacterial colonies were observed after seven days of incubation at 30 °C. Furthermore, the ability of the strains to solubilize inorganic tri-calcium phosphate was quantitatively assessed using the malachite green method as described previously^14^.

### Siderophore production

Chrome azurol sulfonate (CAS) agar solid medium was used to screen siderophore production by the rhizobacterial isolates^34^. The reaction was considered positive when an orange halo surrounding the bacterial colony appeared due to the removal of iron from CAS by the siderophore. For the quantitative estimation of siderophore, CAS-shuttle assay was performed following the protocol described previously^28^. Briefly, the quantification of siderophore production was estimated by following the formula: % siderophore unit = [(Ar−As)/Ar] × 100, where, Ar = absorbance of reference at 630 nm (minimal medium + CAS assay solution), and As = absorbance of the sample at 630 nm.

### IAA production

Production of indole-3-acetic acid (IAA) by the rhizobacterial isolates was estimated by growing the isolates in M9 medium, supplemented with or without L-tryptophan (100 µg ml^−1^), and incubated for 48 h at 30 °C. After incubation, 1 ml of supernatant was mixed vigorously with 4 ml of Salkowski's reagent (150 ml conc. H_2_SO_4_, 250 ml H_2_O, 7.5 ml 0.5 M FeCl_3_. 6H_2_O), incubated for 30 min and the absorbance was measured at 520 nm with the help of a spectrophotometer (Halo XB-10 by Dynamica Scientific Ltd., United Kingdom). The concentration of IAA produced by the rhizobacterial isolates was determined from a standard curve generated using a standard solution of commercial IAA (Sigma–Aldrich, Germany).

### Ammonia production

To detect the production of ammonia, the rhizobacterial isolates were grown in 4% peptone broth and incubated for seven days at 30 °C. Following incubation, 0.5 ml of Nessler’s reagent was added to the bacterial suspension. The development of brown to yellow color indicates ammonia production. The absorbance was measured at 450 nm using a spectrophotometer. Furthermore, quantitative estimation of the amount of ammonia production by the rhizobacterial isolates was carried out and compared with a standard curve generated using a standard ammonium sulphate solution.

### Effect of the isolates on the growth of fungal cultures

To assess whether the isolates exhibit any antifungal activity, their effects on the growth of different fungal cultures were measured using dual-culture technique^35^. On a single potato dextrose agar (PDA) plate, both the fungal strain and an individual isolate were inoculated on two halves. These PDA plates were then incubated at 30 °C for three days. The effect of each isolate on the growth of the fungal culture was then assessed by measuring the spread of the culture towards the bacterial colony. The less the spread is, the more the antifungal effect of the isolate is. Two different fungal cultures, *Rhizoctonia solani* AG1-IA and *Ustilago maydis* SG200, a necrotrophic and a biotrophic phytopathogen respectively, were used in this assay.

### Protease activity

The protease activity of the bacterial isolates was determined using the skim milk agar medium as described previously^25^. The isolates were spot inoculated on skim milk agar medium, and after two days of incubation at 30 °C, proteolytic activity was assessed by clear zone around the colonies.

### Cellulase activity

The rhizobacterial isolates were screened for cellulase production by plating them onto M9 agar media supplemented with 10 g l^−1^ carboxymethyl cellulose (CMC) (Sigma-Aldrich, Germany). After 48 h incubation at 30 °C, plates were flooded with congo red dye, and the clear halos formed surrounding the colonies indicated their cellulolytic activity^36^.

### ACC deaminase activity

To detect whether the rhizobacterial isolates were able to produce the ACC deaminase enzyme, the organisms were streaked onto M9 minimal media containing 1-aminocyclopropane-1-carboxylic acid (ACC) as the sole source of nitrogen. The plates were incubated at 30 °C for 48–72 h. An abundant growth on the medium indicated the positive result for a corresponding rhizobacterial isolate.

### Effect of rhizobacterial isolates on the growth of rice and maize seedlings

To assess the effect of rhizobacterial isolates on rice seedlings, surface-sterilized rice seeds (Variety: IR64) were sown in sterile pots (20–25 seeds/pot) [Pot size (L × W × H): 20 cm × 20 cm × 16 cm] filled with sterilized soilrite (Keltech Energies Limited, Bangalore, India) and allowed to germinate in the dark for about five days. Following germination, the pots were transferred to light, and the rhizosphere of the rice seedlings was treated with freshly grown cultures of about 10^8^–10^9^ cfu ml^−1^ of individual rhizobacterial isolates^14,37^. Rice seedlings treated with the sterile growth media for the culture of the rhizobacterial isolates were used as a negative control. One rhizobacterial isolate was tested in 16 different pots. Four of these 16 pots were used for the experiment of assessing the effect of individual rhizobacterial isolates on the growth of rice seedlings. Different growth parameters like the root length, shoot length, dry weight, and wet weights of 4 randomly selected rice seedlings from each of the four pots were measured at 21 days post-treatment and compared with that of the control seedlings. These 16 rice seedlings in total (four from each of the four pots) represented 16 replicates of the experiment. For the measurement of chlorophyll content following 21 days post-treatment, 16 rice seedlings were randomly selected from the same four pots, as mentioned in the case of growth parameter assessment experiment. The seedlings in the remaining 12 pots were used at 14 days post-treatment for assaying the antioxidative enzymatic activities, the total contents of proline and phenols in three sets of four pots each. The plants were regularly irrigated. Both the control and treated seedlings were maintained in a phytochamber in a 16 h/8 h light/dark cycle. Day and night temperatures were maintained at 30 °C and 24 °C, respectively, with average humidity 70% throughout the experiment.

To assess the effect of rhizobacterial treatment on the growth parameters of maize seedlings (Variety: Early Golden Bantam), a previously described methodology was adopted with certain modifications^14^. Briefly, after germination, rhizospheres of 7-day-old seedlings were treated with freshly grown cultures of about 10^8^–10^9^ cfu ml^−1^ of individual rhizobacterial isolates. Similar to rice, each of the rhizobacterial isolates were tested for growth promotion in 16 replicates. In total, 16 pots were used for the treatment of each of the rhizobacterial isolate, each pot containing two maize seedlings. While one of the treated seedlings in each pot was used for assessing different growth parameters like shoot length, root length, dry weight, and wet weight, the other seedling was used to assess the total chlorophyll content following 15 days post-treatment. Control and treated maize plants were grown in a phytochamber in a 15 h/9 h light/dark cycle, and the temperature was maintained at 28 °C and 20 °C with relative humidity 40% and 60% during light and dark periods, respectively.

### Measurement of chlorophyll

Chlorophyll a, and chlorophyll b contents were measured in leaves of soil rite grown, and PGPR inoculated rice and maize plants. Chlorophyll pigments were extracted from fresh leaf samples in 80% acetone following the procedures described previously^38^, and the quantification was carried out following the method described by Porra et al.^39^.

### Assessment of antioxidative enzymes, proline, and total phenol contents in treated rice plants

To evaluate the triggering of antioxidative defense response in rice plants upon PGPR treatment, a series of plant defense-related enzymatic assays and estimation of non-enzymatic molecules were performed. Plant samples either treated with growth media only or individual rhizobacterial isolates were collected 14 days post-treatment and washed adequately to remove traces of soil rite. Following this, the plant samples were crushed in liquid nitrogen and stored at − 80 °C till further use.

### Plant defense-related enzyme assay

Root and shoot proteins were extracted from frozen crushed samples in 3 mL of extraction buffer containing 100 mM K-phosphate buffer (pH 7.0), 2 mM EDTA, 20 mM ascorbate, and 0.1% (v/v) Triton X-100 for catalase (CAT) and ascorbate peroxidase (APX) activities or 100 mM Citrate–phosphate buffer (pH 5.5) for chitinase or 100 mM Na-Borate buffer (pH 8.8) containing 5 mM β- mercaptoethanol, 2 mM EDTA and 10% acid-washed polyvinylpolypyrrolidone (PVP) for Phenylalanine ammonia-lyase (PAL). The homogenate was then centrifuged at 12,000 × *g* for 20 min at 4 °C. The supernatant was used for total protein and enzymatic assays.

Root and shoot ascorbate peroxidase (APX) activities were measured at 290 nm following a method described previously^40^. 20 μL enzyme extract was added to a 3 mL APX assay mixture containing 50 mM K-phosphate buffer (pH 7.0), 0.1 mM H_2_O_2_, and 0.5 mM ascorbate. The amount of ascorbate oxidized was calculated using the extinction coefficient = 2.8 mM^−1^ cm^−1^. All assays were performed in triplicate.

The total root and shoot catalase (CAT) activities were estimated based on the rate of H_2_O_2_ consumption at 240 nm^41^. The assay mixture of 3 mL contained 100 mM K-phosphate buffer (pH7.0), 0.1 mM EDTA, 0.1% H_2_O_2_, and 20 μL enzyme extract. After addition of the enzyme extract to the reaction mixture, a decrease in H_2_O_2_ levels was measured at 240 nm with a Halo XB-10 spectrophotometer and quantified by using the extinction coefficient (0.036 cm^2^ µmol^−1^). All assays were performed in triplicate.

Root and shoot chitinase activities were measured following the protocol described previously with slight modifications^42^. Briefly, 200 µl of crude extract was incubated with 400 µl of 100 mM citrate–phosphate buffer (pH 5.5) and 200 µl of 1% colloidal chitin at 37 °C for 1 h in a water bath. The reaction was then arrested by adding 1.5 ml 3, 5-dinitrosalicylic acid (DNS) reagent, followed by heating at 100 °C for 10 min in a boiling water bath. After cooling the reaction mixture to room temperature, the colored solution was centrifuged at 10,000 × *g* for 10 min, and the absorbance of the supernatant was measured at 540 nm using a UV- visible spectrophotometer (Halo XB-10). One unit of the chitinase activity was defined as the amount of enzyme that catalyzed the release of 1 µmol/ml/min of reducing sugar under the assay conditions. All assays were performed in triplicate.

Phenylalanine ammonia-lyase (PAL) activity of the root and shoot extracts were measured following the method described previously^43^. 200 µl of the enzyme extract was preincubated with 30 mM Na-Borate buffer (pH 8.8) at 30 °C for 10 min, and then 100 µM phenylalanine was added as substrate making the total reaction volume of 3 ml. The reaction mixture was then incubated at 37 °C for 70 min. The reaction was stopped with 100 µl of 6 N HCl, and the absorbance was measured at 290 nm using a Halo XB-10 UV–visible spectrophotometer. The specific activity of PAL was defined as the amount of enzyme required for the formation of 1 mol of product in 1 s per mg of protein under the assay conditions.

### Estimation of total proline

Proline was estimated using a method described previously with slight modifications^44^. Briefly, 500 µl of 3% sulfosalicylic acid was added to 100 mg of crushed samples prepared from the PGPR-treated and non-treated control rice plants. The mixture was then centrifuged for 10 min at room temperature at 10,000 × *g*, and the supernatant was used for further analysis. 100 µl of supernatant was mixed with 100 µl of 3% sulfosalicylic acid, 200 µl acid-ninhydrin, and 200 µl of glacial acetic acid in a microfuge tube. The reaction mixture was mixed thoroughly and was further incubated for 1 h at 100 °C. The reaction was terminated by placing the microfuge tube on ice followed by proline extraction with 1 ml toluene. The chromophore-containing layer was collected and kept at room temperature for 15 min before measuring the absorbance at 520 nm using a spectrophotometer. The proline concentration was determined using a standard curve, and the results were expressed as µg/gram fresh weight.

### Total phenolic contents

The total phenolic content (TPC) was estimated using a modified Folin–Ciocalteu method^45^. Briefly, the total phenolics were extracted twice (each for 10 min at 70 °C with occasional mixing) with 50 ml of hot demineralized water from 1.5–2 g of air-dried leaves. 500 μl of extracts in demineralized water was mixed with 2.5 ml of Folin- Ciocalteu reagent (0.2 N). After 5 min, 2 ml of Na2CO3 solution (75 g/l) was added and kept in the dark for 120 min, before measuring the absorbance at 760 nm against a blank using a Halo XB-10 UV–visible spectrophotometer. The total phenolic contents were calculated based on the calibration curve of gallic acid and expressed as gallic acid equivalents (GAE) in milligrams per gram of the sample.

### Evaluation of the effect of multispecies rhizobacterial consortia on the susceptibility of rice plants towards *R. solani* AG1-IA infection

To assess the effect of rhizobacterial consortia, if any, in the disease resistance of host plants, rice seedlings pretreated with rhizobacterial consortia were infected with foliar pathogen *Rhizoctonia solani* AG1-IA that causes sheath blight infection in rice. To develop multispecies rhizobacterial consortia, 30 rhizobacterial isolates were randomly distributed in six different groups, each consisting of five isolates representing different genera/species (Table S1). For the pretreatment of rice seedlings with the rhizobacterial consortia, each of the individual isolates of the consortia is inoculated in 5 ml Luria Bartoni (LB) broth and grown at 30 °C with constant agitation at 160 rpm till an OD_600_ of the culture reached 0.6. Following this, equal volumes of each of the rhizobacterial suspension cultures were mixed to achieve a final concentration of 10^8^–10^9^ cfu ml^−1^. 1 ml of the resulting culture was used to treat the rhizospheric region of the five days old IR64 variety of rice seedlings. The treated plants were grown at 24 °C under 16 h/8 h day/night cycle for 3 days. Following this, the seedlings were inoculated with *R. solani* AG1-IA. The inoculation was done by incubating about 1 cm length of the rice seedlings with potato dextrose agar plaques containing both sclerotia and mycelia of *R. solani* AG1-IA. The rice seedlings thus infected with the fungus were grown further for two days following which the infection symptoms were scored as the length of the chlorotic lesions in the infected area of the leaves. The severity of the infection was then accessed as the disease index (DI). The DI is the measure of the spread of infection beyond 1 cm inoculation area. The average spread of infection in the case of rice seedlings that were untreated with any rhizobacterial consortia (control) was considered as the standard. In the case of all of the rhizobacterial consortia treated rice seedlings (treated), the spread of the infection was presented as the fraction of that of the control. The DI, therefore, in case of the treated *R. solani* AG1-IA infected seedlings was calculated as below:

$${\text{DI}}_{{{\text{treated}}}} = \, \left( {{\text{Total lesion length in cm}}^{{ - {1}}} } \right)_{{{\text{treated}}}} /{\text{Average of }}\left( {{\text{Total lesion length in cm}}^{{ - {1}}} } \right)_{{{\text{control}}}} .$$

The DI of the control is considered as 1. The DI of all the treated samples thus calculated was then compared with that of the control to evaluate the effect of the respective consortia on inducing resistance to disease susceptibility in rice.

### Statistical analysis

For each of the investigated biochemical parameters from control and treated samples, three separate replication sets were conducted. All the experimental measurement values were expressed as means of three measurements [± standard deviation (SD)]. The significance of the differences between the mean values of control and treated plant samples were statistically evaluated by two-tailed t-test at P ≤ 0.05 using the Windows 2016/Microsoft Excel 2013 computer package.

### Nucleotide sequence accession numbers

The partial 16S rRNA gene sequences of the rhizobacterial isolates reported in this study were submitted to GenBank under the accession numbers: MT436081–MT436110.

## Results and discussion

### Isolation and identification of rhizobacteria from tea rhizosphere

Soil microorganisms play a crucial role in plant health and development. Moreover, they contribute immensely to the agricultural production of different crops. In the district of Darjeeling, tea is cultivated as the major cash crop. Besides tea, a number of other crops such as rice, maize, wheat, mustard, millet, ginger, orange, large cardamom, and vegetable crops are cultivated^19^ (Source: https://darjeeling.gov.in/agriculture.html). Rice and maize are the most important food grain crops grown in this region. However, because of the acidic nature of the soil of this region, crop cultivation becomes increasingly difficult. Agrochemicals, including N fertilizers, make the situation even more complicated as they further assist soil acidification. In the slightly acidic soils of Darjeeling district (4.2 < pH < 7.0), base cations such as calcium (Ca), magnesium (Mg), potassium (K) and sodium (Na) are replaced by protons and subsequently leached from the rhizosphere zone of the crops and thereby become unavailable^22^. Previous studies have shown that the lower soil availability of Ca, Mg, K, and P can be detrimental towards crop growth and development in acidic soils^46,47^. Moreover, crop production is shown to be already limiting at pH values below 5.5–6.5^48^. Under these agroclimatic conditions, plant growth-promoting rhizobacteria offer useful alternatives to agrochemicals for better growth and development of crop plants by direct as well as indirect mechanisms^2^. Tea rhizosphere harbors diverse plant growth-promoting rhizobacteria, which offer potential applications as biofertilizers in sustainable agricultural practice within a similar agroclimatic setup^12–16,18,49^. We, therefore, sought to isolate, characterize, and explore tea PGPR for their potential in plant growth promotion and biocontrol using the two most important food grain crops in the Darjeeling region.

In the present study, a total of 120 unique rhizobacteria were isolated from the soil samples collected from seven tea estates of Darjeeling, West Bengal, India (Fig. 1) using different enrichment media. All the rhizobacterial isolates were then functionally screened on specific media for assessing their plant-growth-promoting (PGP) potential, and eventually, thirty pure cultures of rhizobacteria were selected for further analysis. At the time of biochemical and microbiological studies, sixteen isolates were found to be *Bacillus* (16 different strains)-like. All of them were found to be spore-forming, Gram-positive, catalase-positive, and rod-shaped bacteria (data not shown). Also, several gram-positive, spherical (grape-like appearance under the microscope) shaped *Staphylococcus*-like bacterial isolates were evident from microbiological analyses (data not shown). In general, the microbiological assessment of the tea rhizosphere soil revealed a bacterial count ranged between 2 × 10^4^ to 2 × 10^7^. The number of rhizobacterial strains isolated from rhizosphere soils of each tea estates is summarized in Table 1 along with soil pH and texture as measured following USDA methodologies.

**Figure 2:**
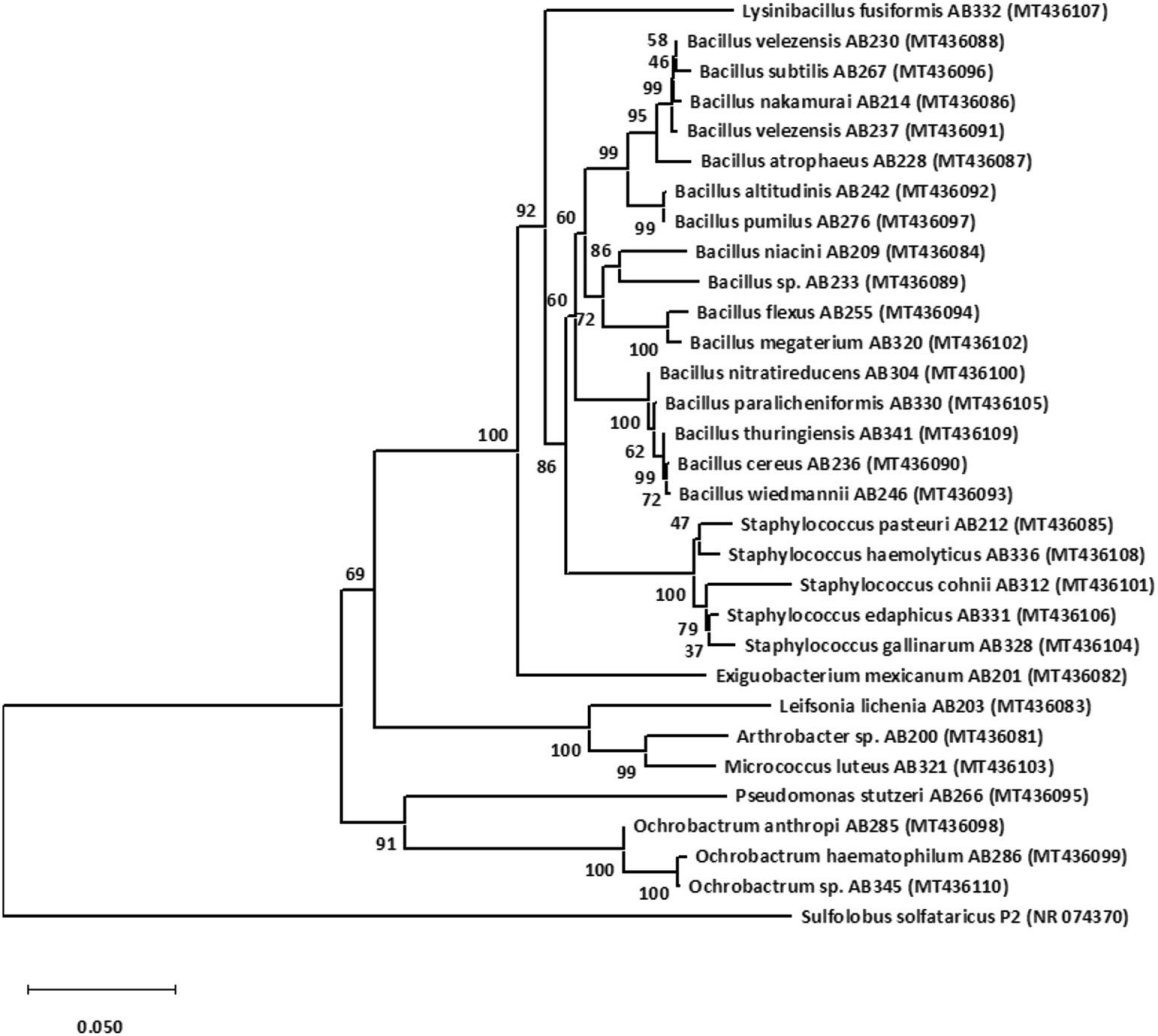
Neighbor-joining phylogenetic tree showing phylogenetic relationship between rhizobacterial isolates based on the 16S rRNA gene sequences. The 16S rRNA sequence of *Sulfolobus solfataricus* P2 was used to assign an outgroup species.

To determine the identity of the tea rhizobacterial isolates, the 16S rRNA gene was amplified partially and sequencing was performed as described in the method section. DNA sequencing and analysis using the BLAST tool revealed that all the 30 rhizobacterial isolates show an identity ranging from 97.88% to 100% with the available 16S rRNA gene sequences in GenBank (Table 2). Furthermore, the phylogenetic analysis revealed that these 30 rhizobacterial isolates represent nine different genera (Fig. 2). Out of these 30 isolates, 16 (53.3%) isolates belong to genus Bacillus, 5 (16.6%) represent genus Staphylococcus, 3 (10%) represent genus Ochrobactrum, and 1 (3.3%) isolates each belongs to genera Pseudomonas, Lysinibacillus, Micrococcus, Leifsonia, Exiguobacterium, and Arthrobacter (Table 2 and Fig. 2). Previous studies have shown that the cultivable rhizobacterial population in the tea rhizosphere is dominated by Bacillus genera^12,14,15,49^. Moreover, Bacillus genera are a dominant cultivable member in the rhizosphere soil of different plants, e.g., rice, wheat, tobacco, *Panax notoginseng* (Chinese ginseng), etc.^50–53^.

**Table 2:** 16S rRNA gene based molecular identity of isolated tea rhizobacteria, their sequence accession numbers and their isolation sites.

Serial number	Strain name	Accession number	Base pair length	Nearest neighbor and % identity from NCBI with Accession number	Isolation source
1	AB200	MT436081	1,321	*Arthrobacter* sp. 690 (99.7%) (EU086821)	Long view tea estate
2	AB201	MT436082	1,427	*Exiguobacterium mexicanum* (100%) (CP040676)	Maharani tea estate
3	AB203	MT436083	1,353	*Leifsonia lichenia* (98.37%) (H432650)	Happy Valley Tea estate
4	AB209	MT436084	1,415	*Bacillus niacini* (98.23%) (MF177863)	Maharani tea estate
5	AB212	MT436085	1,401	*Staphylococcus pasteuri* (99.79%) (MT072161)	Long view tea estate
6	AB214	MT436086	1,414	*Bacillus nakamurai* (99.93%) (MH057388)	Barnesbeg tea estate
7	AB228	MT436087	1,416	*Bacillus atrophaeus* (98.45%) (KP209406)	Makai Bari Tea estate
8	AB230	MT436088	1,399	*Bacillus velezensis* (99.93%) (MT378136)	Maharani tea estate
9	AB233	MT436089	1,417	*Bacillus* sp. (99.72%) (KP217809)	Rohini Tea estate
10	AB236	MT436090	1,413	*Bacillus cereus* (99.65%) (KU922345)	Tukvar tea estate
11	AB237	MT436091	1,349	*Bacillus velezensis* (100%) (MH157241)	Makai Bari Tea estate
12	AB242	MT436092	1,410	*Bacillus altitudinis* (100%) (KX344022)	Barnesbeg tea estate
13	AB246	MT436093	1,405	*Bacillus wiedmannii* (99.86%) (LC515603)	Maharani tea estate
14	AB255	MT436094	1,413	*Bacillus flexus* (100%) (MH542283)	Happy Valley Tea estate
15	AB266	MT436095	1,349	*Pseudomonas stutzeri* (99.33%) (MN932299)	Barnesbeg tea estate
16	AB267	MT436096	1,411	*Bacillus subtilis* (99.86%) (LC040931)	Tukvar tea estate
17	AB276	MT436097	1,359	*Bacillus pumilus* (100%) (MK603127)	Long view tea estate
18	AB285	MT436098	1,255	*Ochrobactrum anthropi* (100%) (MT093466)	Rohini Tea estate
19	AB286	MT436099	1,346	*Ochrobactrum haematophilum* (100%) (MH236269)	Makai Bari Tea estate
20	AB304	MT436100	1,355	*Bacillus nitratireducens* (100%) (MT341781)	Long view tea estate
21	AB312	MT436101	1,390	*Staphylococcus cohnii* (97.88%) (MN581170)	Makai Bari Tea estate
22	AB320	MT436102	1,332	*Bacillus megaterium* (99.92%) (CP010586)	Rohini Tea estate
23	AB321	MT436103	1,366	*Micrococcus luteus* (99.93%) (AB539843)	Barnesbeg tea estate
24	AB328	MT436104	1,419	*Staphylococcus gallinarum* (100%) (MK015788)	Barnesbeg tea estate
25	AB330	MT436105	1,401	*Bacillus paralicheniformis* (100%) (MG780252)	Maharani tea estate
26	AB331	MT436106	1,255	*Staphylococcus edaphicus* (100%) (MT269536)	Tukvar tea estate
27	AB332	MT436107	1,362	*Lysinibacillus fusiformis* (98.07%) (LT547806)	Happy Valley Tea estate
28	AB336	MT436108	1,400	*Staphylococcus haemolyticus* (100%) (MN581168)	Rohini Tea estate
29	AB341	MT436109	1,373	*Bacillus thuringiensis* (99.56%) (MN911366)	Makai Bari Tea estate
30	AB345	MT436110	1,296	*Ochrobactrum* sp. (98.84%) (KY678891)	Maharani tea estate

### In vitro plant-growth promoting activities

Thirty selected rhizobacterial isolates were evaluated in vitro for properties that are known to be essential for plant growth-promoting activities of bacteria, such as the IAA production, phosphate solubilization, siderophore production, and ammonia production.

Plant growth-promoting rhizobacteria often produce indole acetic acid (IAA) and thereby assist plant growth. The biosynthesis of bacterial IAA takes place either by tryptophan-dependent or independent manners. Out of 30 rhizobacterial isolates, 21 (70%) showed IAA production of the level of 20 µg ml^−1^ or more when grown in the presence of 100 µg ml^−1^ L-tryptophan (Table 3). Some of the rhizobacterial isolates (AB304, AB312, AB328, and AB331) were found to synthesize 90 µg ml^−1^ or more of IAA with AB331 synthesizing the maximum amount (134.67 ± 3.59 µg ml^−1^) (Table 3). Biosynthesis of IAA utilizing tryptophan has been reported previously in different PGPR strains^54^. Previous studies have shown that tea rhizobacteria are efficient in IAA production in the presence of tryptophan^15,16^. However, in the present study, we also determined rhizobacterial IAA production in the absence of tryptophan. In the absence of tryptophan, 8 (26.6%) isolates showed 20 µg ml^−1^ or more IAA production with AB304 being the highest (55.17 ± 5.42 µg ml^−1^) (Table 3).

**Table 3:** Functional screening of selected tea rhizobacteria for in vitro PGP and antifungal activities.

Sl No	Sample	Plant growth promoting traits					Antifungal traits			
		IAA production (µg ml^−1^)*	IAA production when supplemented with Tryptophan (µg ml^−1^)*	Siderophore production (%)*	Phosphate solubilization (µg ml^−1^)*	Ammonia production^#^	Protease production (qualitative)	Cellulase production (qualitative)	ACC deaminase (qualitative)	Anti fungal activity against *R. solani*
1	AB200	6.67 ± 2.86	15 ± 2.36	97.21 ± 0.56	30 ± 2.52	Moderate	−	−	−	+
2	AB201	1.95 ± .034	7.97 ± 1.03	95.05 ± 1.15	230.52 ± 8.75	Small	+	+	+	−
3	AB203	0.76 ± .19	20.34 ± 1.26	98.88 ± 0.42	175.88 ± 8.42	Small	−	+	−	−
4	AB209	3.67 ± 0.47	14.83 ± 2.63	52.31 ± 4.46	280.18 ± 69.09	Large	+	−	−	+
5	AB212	0.68 ± .024	30 ± 2.71	92.71 ± 2.16	275.58 ± 43.63	Moderate	−	−	−	+
6	AB214	3.33 ± 0.36	16.67 ± 1.43	72.07 ± 3.67	572.62 ± 23.59	Moderate	+	+	−	+
7	AB228	1.36 ± 0.72	9.92 ± 6.11	98.55 ± 1.50	51.99 ± 2.04	Small	−	+	−	+
8	AB230	25.33 ± 2.83	37.33 ± 2.83	97.69 ± 1.49	794.86 ± 12.87	Moderate	+	−	+	−
9	AB233	10.33 ± 1.89	20 ± 2.84	94.77 ± 3.22	187.19 ± 37.26	Small	+	+	−	+
10	AB236	25.33 ± 2.83	56.33 ± 3.33	94.43 ± 1.04	22 ± 2.05	Moderate	+	−	+	−
11	AB237	22 ± 0.47	25.33 ± 1.89	75.05 ± 1.64	635.75 ± 18.89	Large	+	−	−	+
12	AB242	4 ± 1.41	38 ± 3.33	72.56 ± 2.46	12 ± 3.0	Large	−	−	+	−
13	AB246	16.5 ± 0.71	20 ± 0.47	80.25 ± 4.60	591.28 ± 53.44	Moderate	+	−	−	+
14	AB255	7 ± .94	15.33 ± 2.83	99.26 ± 1.21	191.51 ± 8.31	Small	+	−	+	−
15	AB266	9.67 ± 1.41	39.33 ± 1.86	88.18 ± 2.77	70.12 ± 5.92	Small	−	−	+	+
16	AB267	7 ± 2.83	12 ± 2.83	68.77 ± 3.69	335.62 ± 23.59	Small	+	−	−	+
17	AB276	15 ± 2.36	16.67 ± 2.86	97.21 ± 0.56	21.78 ± 5.75	Moderate	−	−	+	+
18	AB285	34.67 ± 4.24	27 ± 3.29	93.25 ± 1.25	229.40 ± 24.67	Small	−	−	+	+
19	AB286	4 ± 1.42	15.67 ± 1.89	82.71 ± 1.25	33.52 ± 4.25	Moderate	+	−	−	−
20	AB304	55.17 ± 5.42	90 ± 4.33	83.87 ± 2.26	137.52 ± 14.41	Large	+	+	−	+
21	AB312	9.17 ± 3.06	113 ± 2.36	99.01 ± 0.21	252.83 ± 9.35	Large	+	−	−	−
22	AB320	28.17 ± 2.12	74.83 ± 5.89	98.21 ± 0.24	452.65 ± 1.28	Moderate	+	+	−	−
23	AB321	13.83 ± 3.06	58.33 ± 2.36	98.92 ± 0.49	446.76 ± 3.84	Moderate	+	−	+	+
24	AB328	15.33 ± 0.47	99.83 ± 2.12	98.42 ± 0.42	577.71 ± 53.83	Moderate	+	−	−	−
25	AB330	26.17 ± 1.18	53 ± 2.65	97.07 ± 0.11	312.37 ± 6.73	Small	+	−	−	+
26	AB331	25.17 ± 3.54	134.67 ± 3.59	93.60 ± 0.07	275.49 ± 4.57	Large	+	+	+	+
27	AB332	19.33 ± 2.36	48.83 ± 2.78	96.38 ± 0.31	522.89 ± 26.27	Large	−	−	+	−
28	AB336	19.67 ± 3.77	30.67 ± 2.71	97.76 ± 0.45	822.39 ± 41.97	Moderate	+	−	−	+
29	AB341	13 ± 4.71	28.50 ± 1.18	93.99 ± 0.41	728.55 ± 71.77	Small	+	−	+	+
30	AB345	9 ± 1.41	60.50 ± 2.42	98.60 ± 0.24	841.42 ± 10.89	Moderate	+	−	−	−

*Mean value (all values are triplicate) ± Standard deviation (SD), + means positive activity and—means no activity.

^#^ Ammonia production attributes are equivalent to Small: 1–3 µmole ml^−1^ , Moderate: 3–6 µmole ml^−1^, and Large: > 6 µmole ml^−1^.

Microorganisms contribute to the natural phosphorous cycling by solubilizing precipitated and fixed phosphorous present in various soil types in a pH-dependent manner. In the acidic soil of the tea plantation, phosphorus is fixed by free oxides and hydroxides of aluminum and iron, resulting in the low availability of soluble phosphate^55^. In the present study, most of the rhizobacterial isolates were found to be efficient phosphate solubilizers and therefore exhibit potentials to be used as plant growth promoters. About 11 (36.6%) isolates exhibited phosphate solubilization of 400 µg ml^−1^ or more with AB345 being the most efficient phosphate solubilizer (841.42 ± 10.89 µg ml^−1^) (Table 3). Previous studies have shown that phosphate solubilization by tea PGPR is accompanied by a lowering of pH in the medium^15,16^. In the present study, we observed a significant decrease in the pH of the medium associated with an in vitro solubilization of phosphate by the selected PGPR. We believe that the production of organic acids by PGPR facilitated the solubilization of the insoluble phosphates. Previously it has been shown that organic acids such as gluconic acid, lactic acid, malic acid, succinic acid, formic acid, citric acid, malonic acid, and tartaric acid are often involved in effective solubilization of the inorganic phosphate^56^.

Selected rhizobacterial isolates were found to be highly efficient producers of siderophore. The siderophore production by the isolates was found to be between 52 to 99% siderophore units (Table 3). About 70% (21 isolates) of rhizobacterial isolates were found to show siderophore production of more than 90% siderophore units or more (Table 3). In general, rhizobacteria require strategies to survive in the highly competitive micro-ecological zone of the rhizosphere. Amongst many strategies adopted by rhizobacteria, biosynthesis of siderophore is one of the critical strategies, where rhizobacteria inhibit the growth of phytopathogenic bacteria or fungi or non-rhizospheric bacteria by depriving them of the essential iron in the rhizosphere microenvironment^57^. Previous studies have reported that tea PGPR from Assam and Darjeeling are efficient in siderophore production, and our results corroborate well with previous findings^15,16^.

Ammonia production by PGPR is one of the essential traits linked to plant growth promotion. In general, ammonia produced by PGPR has been shown to supply nitrogen to their host plants and thereby promote root and shoot elongation and their biomass^58^. In the present study, the ammonia production by the rhizobacterial isolates was observed in the range of 2.5 μmol ml^−1^ to 7.54 μmol ml^−1^ (Table 3). About 67% (20 isolates) showed ammonia production of more than 4 μmol ml^−1^, and isolate AB331 was found to produce the highest amount of ammonia (7.54 μmol ml^−1^).

Together, the selected tea PGPR isolates were found to be highly efficient in various in vitro PGP activities and showed possibilities in in-planta growth promotion activities.

### Anti-fungal (antagonistic) activities

The application of PGPR to mitigate biotic stress has attracted considerable attention in recent years. Such an application can help both in growth promotion as well as in disease control within the host plant, thereby increasing crop productivity to meet the global demand. All the selected rhizobacterial isolates were screened for antifungal activity against two fungal pathogens, viz. rice necrotrophic pathogen *R. solani* AG1-IA and maize biotrophic pathogen *U. maydis* SG200, respectively. Out of 30 selected rhizobacteria, 18 (60%) isolates were found to be active against *R. solani* AG1-IA (Table 3), and 21 (70%) isolates were active against *U. maydis* SG200 (Data not shown). Seventeen rhizobacterial isolates were found to show activity against both the fungal pathogen tested in the present study. All 30 isolates were further evaluated for protease, cellulase, and ACC deaminase activities. While different hydrolytic enzymes such as proteases and cellulases help in biocontrol by promoting fungal cell wall degradation, ACC deaminase produced by rhizobacteria promotes plant growth by sequestering and cleaving 1-aminocyclopropane-1-carboxylate (ACC) produced in plants under biotic and abiotic stresses^59,60^. Among the isolates, 21 (70%) showed protease activity, 8 (26.6%) showed cellulase activity, and 12 (40%) exhibited ACC deaminase activity (Table 3). To this end, most of the selected rhizobacterial isolates possess the necessary arsenal to act as possible biocontrol agents. We, therefore, sought to test their ability to act as biocontrol agents in our subsequent experiments.

### Assessment of plant-growth promotion activity under laboratory conditions

**Figure 3:**
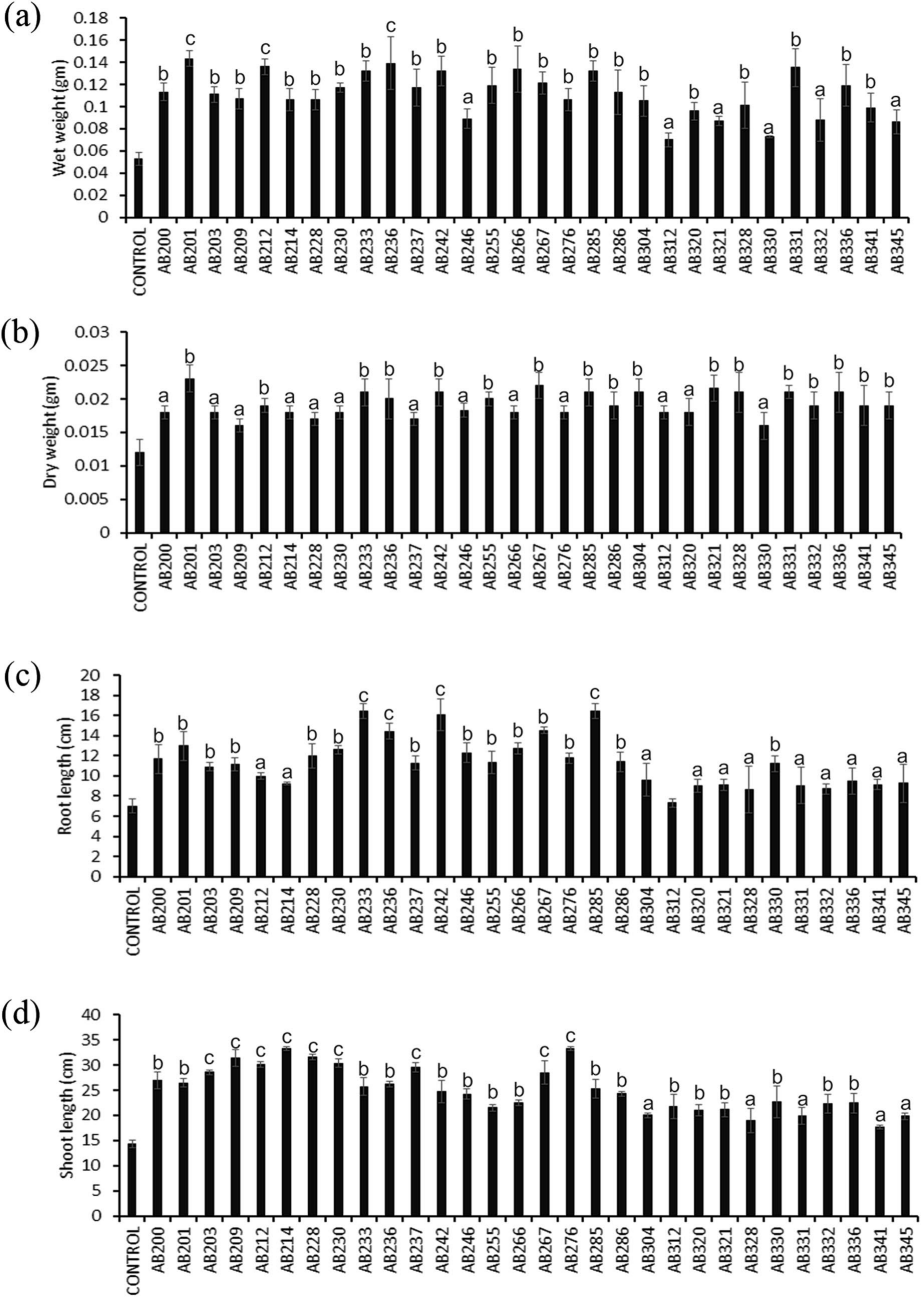
Evaluation of plant-growth promoting traits upon individual treatments of selected tea rhizobacterial isolates on IR64 variety of rice seedlings. Five days old seedlings were treated with individual rhizobacterial isolates, and the growth parameters such as (**a**) wet weight, (**b**) dry weight, (**c**) root length, and (**d**) shoot length were measured 21 days post-treatment. A two-sided t-test determined the significance level, and data are mean ± SD. (**a**: P-value- 0.05–0.01, **b**: P-value 0.01–0.001, and **c**: P-value less than 0.001).

**Figure 4:**
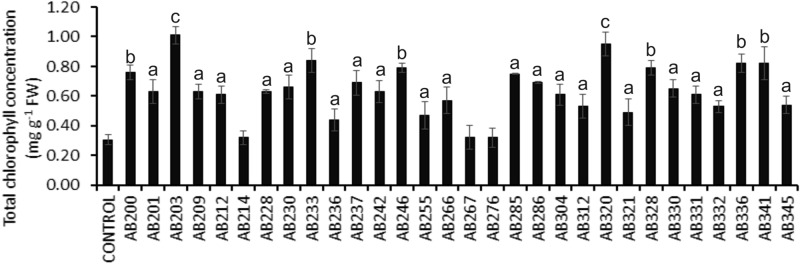
Evaluation of Chlorophyll concentration upon individual treatments of selected tea rhizobacterial isolates on the IR64 variety of rice seedlings. Five days old seedlings were treated with individual rhizobacterial isolates, and the total chlorophyll concentration was measured 21 days post-treatment. A two-sided t-test determined the significance level, and data are mean ± SD. (a: P-value- 0.05–0.01, b: P-value 0.01–0.001, and c: P-value less than 0.001).

The Plant growth promotion experiments using rhizobacterial isolates revealed that the application of almost all the selected rhizobacteria had increased plant biometric parameters (viz. wet weight, dry weight, shoot length and root length) of both rice and maize seedlings in a statistically significant manner (Fig. 3 and Fig. S1). In the present study, Bacillus was the most abundant member of the cultivable tea rhizobacterial isolates. All the 16 (53.33%) isolates of Bacillus genera were found to induce a significant increase in wet weight, dry weight, shoot length and root length in both rice and maize seedlings (Fig. 3 and Fig. S1). Several species of the genus Bacillus such as *B. amyloliquefaciens*, *B. aryabhattai*, *B. circulans*, *B. coagulans*, *B. licheniformis*, *B. megaterium*, *B. subtilis*, *B. thuringiensis* and *B. velezensis* have been previously identified and characterized as PGPR and biocontrol agents^1,12,14,61,62^. They promote plant growth using various direct and indirect mechanisms, including nitrogen fixation, phosphate and potassium solubilization, phytohormone production, siderophores production, antimicrobial and hydrolytic enzymes biosynthesis, stimulation of induced systemic resistance (ISR) and antioxidative defense system in plants^62^. In the present study, we evaluated plant growth-promoting activities of several known PGPR of the genus Bacillus, i.e., *B. atrophaeus* AB228, *B. velezensis* AB230, *B. velezensis* AB237, *B. cereus* AB236, *B. altitudinis* AB242, *B. wiedmannii* AB246, *B. flexus* AB255, *B. subtilis* AB267, *B. nitratireducens* AB304, *B. magatarium* AB320, *B. thuringiensis* AB341 (Fig. 3 and Fig. S1)^61^. Besides, we also found several new members that fall under the genus Bacillus, e.g., *B. niacini* AB209, *B. nakamurai* AB214, *Bacillus* sp. AB233, *B. pumilus* AB276, and *B. paralicheniformis* AB330, as potential plant growth stimulating rhizobacterial isolates (Fig. 3 and Fig. S1). Previous studies have shown that the genus Bacillus dominates the cultivable portion of the Darjeeling tea rhizobacterial population and are useful as PGPR and biocontrol agents^15,63^. In this study besides Bacillus, members of the genus such as Arthobacter (*Arthrobacter* sp. AB200), Exiguobacterium (*Exiguobacterium mexicanum* AB201), Leifsonia (*Leifsonia lichenia* AB203), Lysinibacillus (*Lysinibacillus fusiformis* AB332), Micrococcus (*Micrococcus luteus* AB321), Ochrobactrum (*Ochrobactrum anthropi* AB285, *Ochrobactrum haematophilum* AB286, *Ochrobactrum haematophilum* AB345), Pseudomonas (*Pseudomonas stutzeri* AB266), and Staphylococcus (*Staphylococcus pasteuri* AB212, *Staphylococcus cohnii* AB312, *Staphylococcus gallinarum* AB328, *Staphylococcus saprophyticus* AB331, *Staphylococcus haemolyticus* AB336) were also found to increase plant biometric parameters (viz. wet weight, dry weight, shoot length and root length) in both rice and maize seedlings (Fig. 3 and Fig. S1). To our knowledge, among these isolates, members of the genus Arthrobacter (*Arthrobacter* sp. AB200), Exiguobacterium (*Exiguobacterium mexicanum* AB201), Leifsonia (*Leifsonia lichenia* AB203), and Lysinibacillus (*Lysinibacillus fusiformis* AB332) were isolated from the tea rhizosphere of Darjeeling and tested for PGP activities for the first time. Out of thirty rhizobacterial isolates, a treatment with 27 (90%) isolates resulted in a statistically significant increase in total chlorophyll content both in rice and maize seedling in comparison to uninoculated control plants (Fig. 4 and Fig. S2).

### Effect of PGPR treatment on the defense-related enzymes in rice

**Figure 5:**
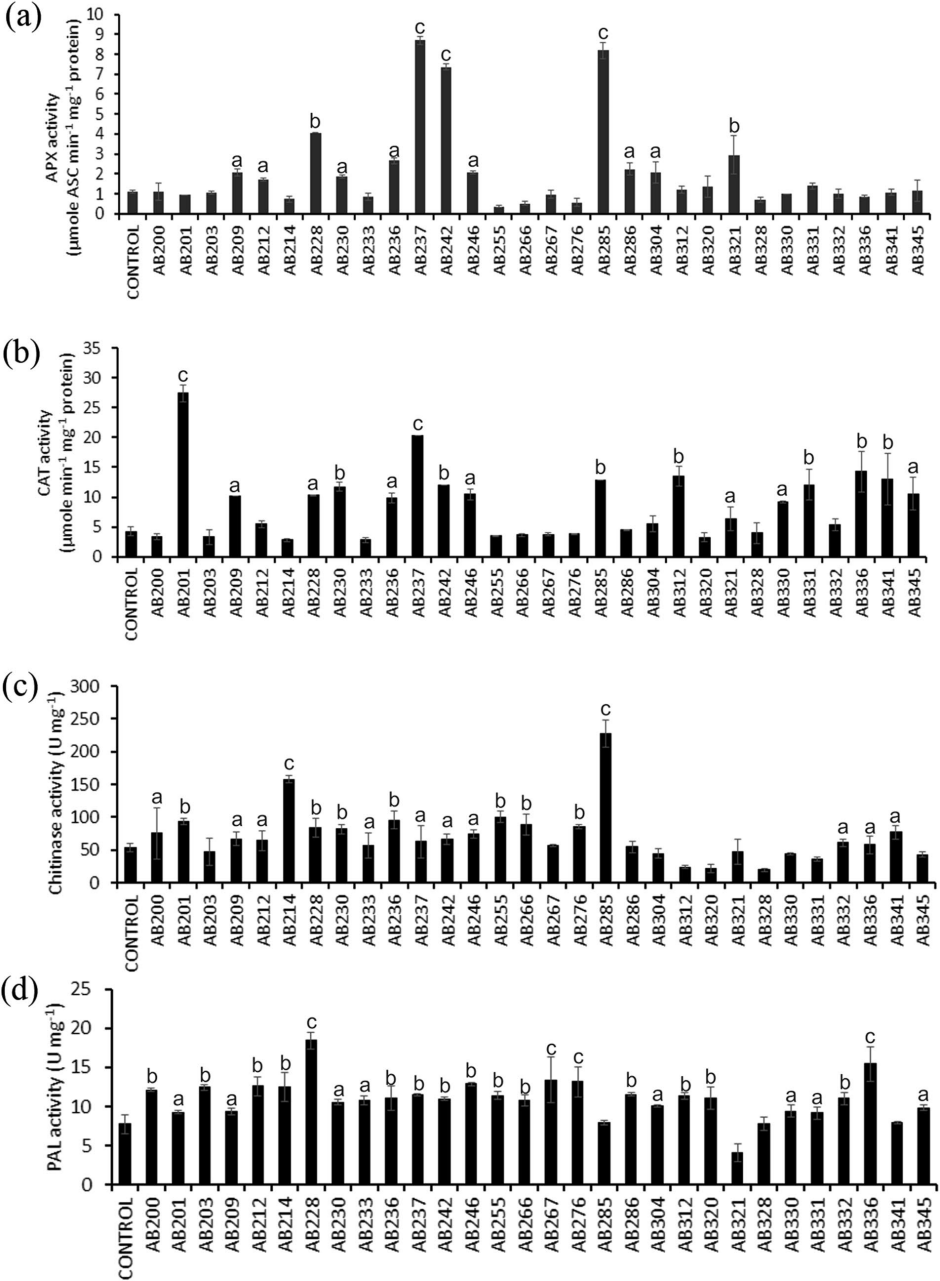
Effect of rhizobacterial treatment on (**a**) ascorbate peroxidase (APX), (**b**) catalase (CAT), (**c**) chitinase, and (**d**) phenylalanine ammonia-lyase (PAL) activity in shoot fraction of IR64 variety of rice seedlings. Five days old rice seedlings were treated with individual rhizobacterial isolates, and the antioxidative defense enzymes were measured in the shoot lysate preparation 14 days post-treatment. A two-sided t-test determined the significance level, and data are mean ± SD. (a: P-value- 0.05–0.01, b: P-value 0.01–0.001, and c: P-value less than 0.001).

Plant growth-promoting rhizobacteria (PGPR) are known to impart induced systemic resistance (ISR) to bacterial, fungal, and viral diseases in plants^64^. They have been shown to elicit plant defense systemically against foliar and root pathogens^65,66^. Previous investigations revealed that different PGPR strains protect the plants from various pathogens by activating plant defense genes encoding chitinase, β-1,3 glucanase, PAL (phenylalanine ammonia-lyase), CAT (Catalase), APX (Ascorbate peroxidase), POD (peroxidase) and other enzymes, many of which act as primary reactive oxygen species (ROS) scavengers^67^. In the present study, we examined the status of APX, CAT, Chitinase, and PAL activities in the PGPR treated rice plants (Fig. 5 and Fig. S3).

The enzyme ascorbate peroxidase detoxifies H_2_O_2_ generated as a byproduct of antioxidative mechanisms and converts it into water within chloroplast, cytoplasm, and mitochondria^9,68^. Increased activity of APX is proposed to have a contribution in the detoxification of augmented H_2_O_2_ accumulation in the cells^63^. Plants inoculated with tea rhizobacterial isolates showed a differential APX activity in the shoots and roots, respectively. In the shoot lysate, APX activity was significantly increased in plants treated with 12 rhizobacterial isolates (40%) (P ≤ 0.05–0.001) (Fig. 5a). In cases of isolates AB237, AB242, and AB285, the APX activity in the shoot lysate were found to be maximum among the treated plants (P ≤ 0.001) (Fig. 5a). In the case of root lysate, significant enhancement of APX activity was evident for 13 rhizobacterial isolates (43.3%) (P ≤ 0.05–0.001) (Fig. S3a). Treatment with isolates AB212, AB236, AB246, and AB255 were found to show maximum APX activity in the root lysates of the treated plants (P ≤ 0.001) (Fig. S3a). Together, APX activity measurements revealed that about 40% and 43.3% rhizobacterial isolates showed statistically significant enhancement of APX activity in the shoot and/or root of the treated plants.

Catalase (CAT) acts as a cellular sink of H_2_O_2_ and catalyzes its disproportionation into H_2_O and O_2_^69^. Out of 30 rhizobacterial isolates, 16 isolates (53.3%) showed a statistically significant increase in the elicitation of CAT activity in plant shoot lysates compared to untreated control (P ≤ 0.05–0.001) (Fig. 5b). Among these isolates, AB201 and AB237 were found to induce maximum CAT activity in plant shoot (P ≤ 0.001) (Fig. 5b). While in the root lysates, CAT activity was found to be significantly higher in the plants treated with 14 rhizobacterial isolates (46.6%) (P ≤ 0.05–0.001) (Fig. S3b). Among these isolates, treatments with AB237 showed a maximum increase in CAT activity in plant root (P ≤ 0.001) (Fig. S3b). Together, the CAT activity was found to increase significantly in the shoot and root of treated plants for treatments with about 53.3% and 46.6% of rhizobacterial isolates.

Chitinases are the member of pathogen-related proteins in plants. These enzymes are strongly induced when a plant is challenged with a fungal pathogen or due to stimulation of induced systemic resistance (ISR) as a result of PGPR–plant interaction^5,70^. Chitinases act as an essential arsenal to mitigate fungal infection in plants by direct lytic action on fungal cell walls or by stimulating a variety of plant defenses by releasing oligosaccharide signaling molecules^70^. Among the selected rhizobacteria, 19 isolates (63.3%) showed a significant increase in chitinase activity in the shoot of treated rice plants (P ≤ 0.05–0.001) (Fig. 5c). Treatment with isolates AB214 and AB285 resulted in statistically most significant increase in the chitinase activity (P ≤ 0.001) (Fig. 5c). Contrary to shoot, the root lysates of the treated plants showed a substantial escalation of the chitinase activity for treatments with 15 rhizobacterial isolates (50%) (P ≤ 0.05–0.001) (Fig. S3c). Furthermore, treatment with AB228 and AB233 showed maximum induction of chitinase activity in the root of the treated plants (P ≤ 0.001) (Fig. S3c). Overall, when treated with about 63.3% and 50% rhizobacterial isolates, a significant increase of the chitinase activity in the shoot and root of treated plants was evident.

Phenylalanine Ammonia Lyase (PAL) is an important enzyme that helps plants to mitigate different stress conditions^5,67^. PAL offers physiological and structural support to the plants by converting L-phenylalanine to ammonia and trans-cinnamic acid. In rice, it was shown that microbial treatment increase PAL activity and accumulation of polyphenols in the leaves and thereby help to ameliorate stress conditions (drought, salinity, etc.)^67^. In the present study, we observed that in the majority of the cases, rhizobacterial treatment resulted in enhanced activity of PAL enzyme in rice shoot lysates. In cases of 26 rhizobacterial isolates (86.6%), a statistically significant increase in the PAL activity was noted for shoot samples of the treated rice plants (P ≤ 0.05–0.001) (Fig. 5d). The most significant PAL activity was, however, documented in the shoot samples of the plants treated with isolates AB228, AB267, AB276, and AB336 (P ≤ 0.001) (Fig. 5d). In root samples, PAL activity was found to increase for treatments with 16 rhizobacterial isolates (53.3%) (P ≤ 0.05–0.001) (Fig. S3d). Out of rhizobacterial isolates, treatment with AB267, and AB341 showed maximum PAL activity in the root samples of the treated plants (P ≤ 0.001) (Fig. S3d). Together, our analysis revealed that about 86.6% of rhizobacterial isolates increase PAL activity in the shoot regions, while only 53.3% isolates could promote PAL activity in the root region of the treated rice plants.

To the end, increased activity of the defense-related enzymes such as APX, CAT, chitinase, and PAL in the PGPR treated rice plants led us to propose that (i) rhizobacterial treatment can stimulate induced systemic resistance (ISR), a state of enhanced defensive capacity in rice plants, and (ii) the rhizobacterial isolates are capable of modulating defense-related enzyme activity and thereby help the plant to prepare itself for a future challenge with biotic and abiotic stresses. Limited information is available about how PGPR from tea rhizosphere modulate defense pathways in host plants^17,71^. To our knowledge, this is possibly the first report of modulation of defense-related enzyme activities by tea PGPR in parallel to their typical plant growth promoting attributes.

### Proline and polyphenols accumulation in inoculated rice plants

ROS scavenging small metabolites such as carotenoids, phenolics, proline, and tocopherol maintain redox balance in cells during oxidative damage^72^. PGPR treatment enhance proline and polyphenolics concentrations that usually favors ROS scavenging in the plants^73^. In the present study, we measured proline and polyphenolics concentrations in the treated rice plants.

**Figure 6:**
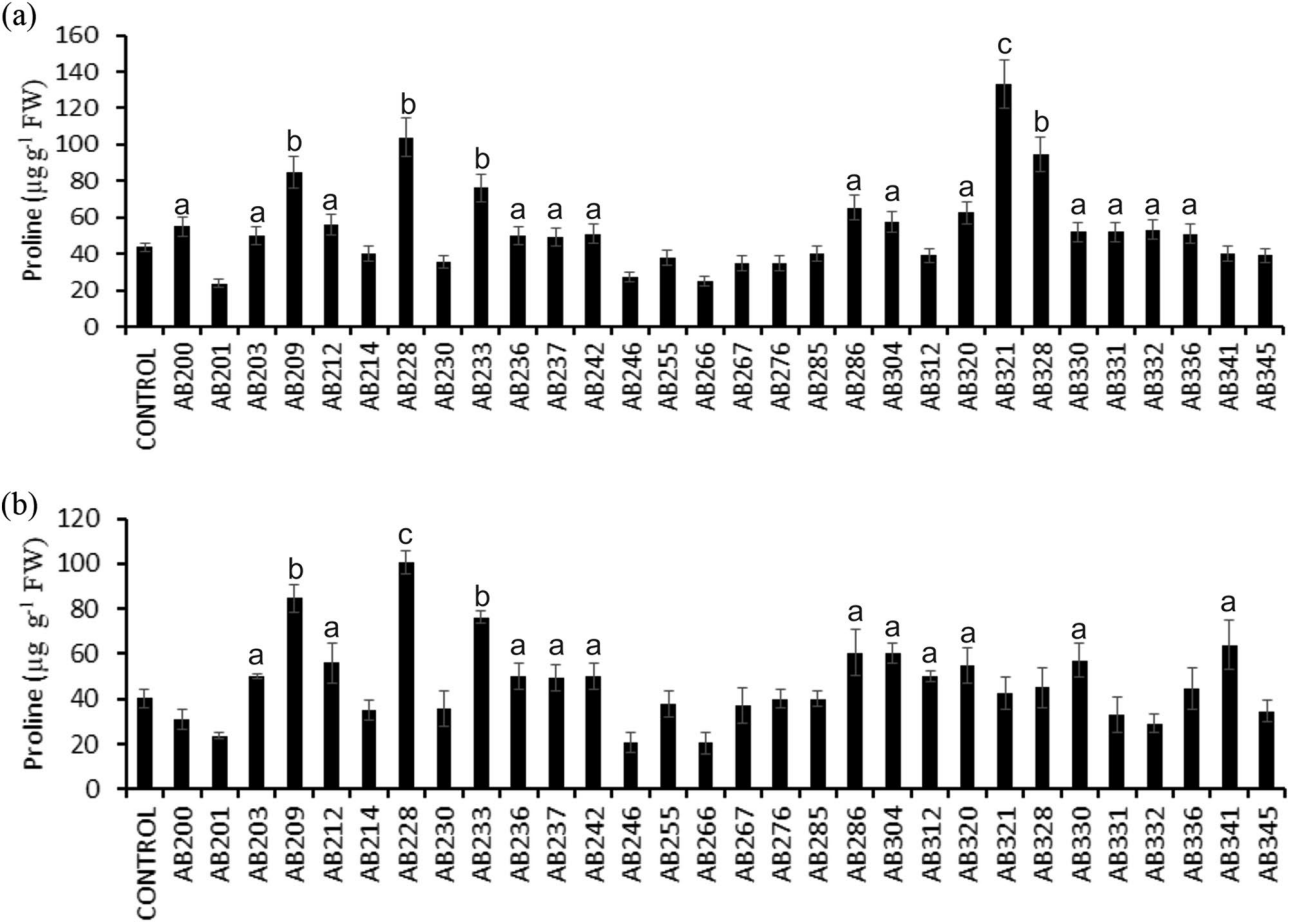
Impact of rhizobacterial treatment on (**a**) shoot proline content, and (**b**) root proline content in IR64 variety of rice seedlings. Five days old rice seedlings were treated with individual rhizobacterial isolates, and the antioxidative defense molecules were measured 14 days post-treatment. A two-sided t-test determined the significance level, and data are mean ± SD. (a: P-value- 0.05–0.01, b: P-value 0.01–0.001, and c: P-value less than 0.001).

Proline is an excellent osmolyte that helps in the stabilization of sub-cellular macromolecules such as proteins and cell membranes. Besides, it is involved in scavenging free radicals, balancing redox homeostasis and signaling, thereby assisting plants to cope under stress conditions^74^. Eighteen rhizobacterial isolates (60%) were found to cause a statistically significant increase in the proline concentration in shoot samples in treated rice compared to untreated control plants (P ≤ 0.05–0.001) (Fig. 6a). The isolates AB321 caused the most significant increase in proline concentration in the shoot of treated rice plants (P ≤ 0.001) (Fig. 6a). In the case of the same set of treatments, analysis of the root samples revealed that 14 rhizobacterial isolates (46.6%) caused a statistically significant increase in proline content (P ≤ 0.05–0.001) with isolate AB228 being the most efficient (P ≤ 0.001) (Fig. 6b). Together, proline estimates in the rhizobacteria treated rice plants revealed that in the majority of the treatments proline concentration was increased in shoot samples (60%) and also in root samples (46.6%) indicating possible PGPR assisted priming (both local and systemic) of the plants to face future challenges of biotic and abiotic stresses.

**Figure 7:**
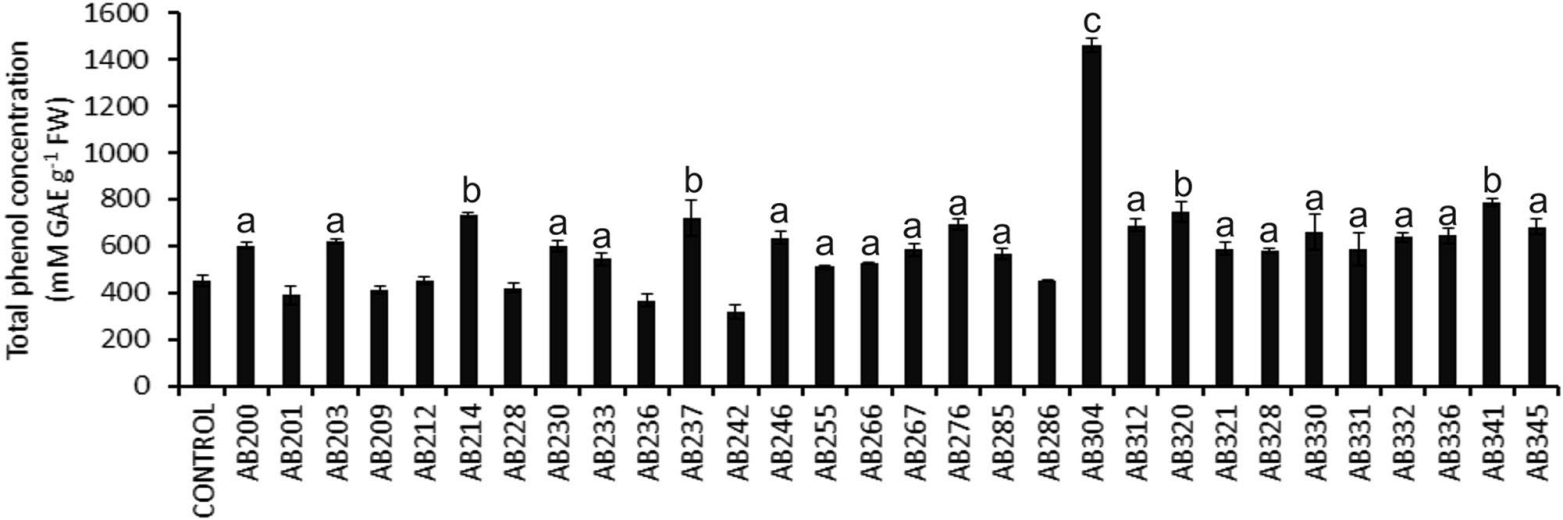
Impact of rhizobacterial treatment on the accumulation of total polyphenol in IR64 variety of rice seedlings. Five days old rice seedlings were treated with individual rhizobacterial isolates, and the antioxidative defense molecules were measured 14 days post-treatment. A two-sided t-test determined the significance level, and data are mean ± SD. (a: P-value- 0.05–0.01, b: P-value 0.01–0.001, and c: P-value less than 0.001).

Accumulation of polyphenolics in plant leaves is shown to have a protective role against biotic and abiotic stresses through anti-oxidation and ROS deactivation^67^. Microbial treatment influences the accumulation of polyphenolics in plant leaves^75^. Being a potent antioxidant, high accumulation of polyphenolics in the leaves is supposed to strengthen plants' stress tolerance^75^. In the present study, we measured the total accumulated polyphenolics in the treated rice plants. Our analysis revealed that for treatments with 23 rhizobacterial isolates (76.6%), a statistically significant increase in total polyphenolics was observed in the leaves (P ≤ 0.05–0.001) (Fig. 7). However, treatment with isolates AB304 caused the most significant effect in terms of total polyphenolics measurement in the leaves (P ≤ 0.001) (Fig. 7). Together, an accumulation of polyphenolics was observed in the majority of the treatments (76.6%), indicating possible enhancement of anti-oxidants in the plants.

### Increased resistance of PGPR pretreated rice plants towards sheath blight infection

**Figure 8:**
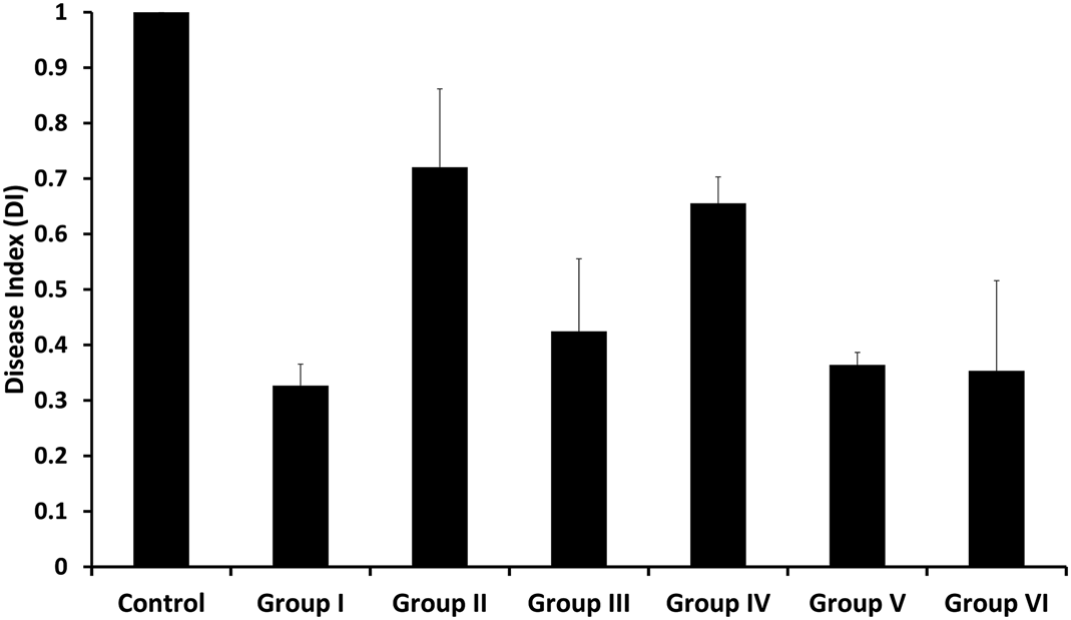
The effect of rhizobacterial treatment on the relative disease indices (DI) of sheath blight infection in IR64 variety of rice seedlings. Five days old rice seedlings were treated with individual rhizobacterial consortia (as described in Table S1), and the treated plants were grown at 24 °C under 16–8 h day-night cycle for three days. Following this, the seedlings were inoculated with *R. solani* AG1-IA, as described in the method section. The rice seedlings thus infected with the fungus were grown further for two days, and the infection symptoms were scored as the disease index (DI). The DI of all the treated samples was calculated and compared with that of the control to evaluate the effect of the respective consortia on inducing resistance of rice seedlings. Error bars represent the standard deviation calculated from three independent experiments.

To assess whether the increased activity of defense-related enzymes in rice due to PGPR pretreatment is indeed involved in inducing disease resistance, we studied sheath blight infection in rice under PGPR pretreated and untreated conditions. PGPR were distributed in six consortia (Table S1), and the rice seedlings were pretreated with each of these six consortia separately before *R. solani* AG1-IA infection. Figure 8 shows a positive response with respect to the increased resistance of rice in the case of each of the six consortia. However, the degree of resistance induced within the rice plants varied with the individual consortia used for pretreatment. Like for instance, groups I, V, and VI showed the maximum effect with about 60 to 70 percent reduction in DI. In all of these cases, the DI ranged between 0.3 and 0.4. Group III showed a moderate effect with a DI of 0.42, and group II and IV showed the least impact. While group IV consortia pretreatment resulted in a DI of 0.65, group II treated rice plants showed a DI of 0.72 upon infection with *R. solani* AG1-IA. Among the designed multispecies rhizobacterial consortia, group I was found to be the most effective in the mitigation of *R. solani* AG1-IA infection. Group I have consisted of *Arthrobacter* sp. AB200, *Staphylococcus pasteuri* AB212, *Bacillus* sp. AB233, *Bacillus altitudinis* AB242, and *Pseudomonas stutzeri* AB266 (Table S1). Out of five rhizobacterial isolates in group I, *Arthobacter* sp. was shown previously to inhibit the in vivo growth of potato pathogen *Phytophthora infestans*^76^, *P. stutzeri* was found effective against plant root rot *Fusarium solani*^77^, and *B. altitudinis* was found to be effective against the root rot disease caused by *Thanatephorus cucumeris*^78^. Besides, groups V and VI were also found highly efficient in the mitigation of *R. solani* infection in rice (Fig. 8). Members of group V, e.g., *B. subtilis*, *B. thuringiensis*, *B. pumilus*, and *S. gallinarum* are well-known biocontrol agents against a number of phytopathogens^79–82^. Group VI members are comparatively less characterized rhizobacterial species (Table S1) and members like *B. paralicheniformis*, *Lysinibacillus fusiformis*, and *Ochrobactrum* sp. were recently reported to have biocontrol activities^83–85^.

## Conclusion

In the present study, thirty plant growth-promoting rhizobacteria were isolated and characterized from the tea rhizosphere of Darjeeling, West Bengal, India. All the thirty rhizobacterial isolates were found to effectively promote the growth of rice and maize seedlings implying their possible utilization in microbe-based bio-formulations. Moreover, the treatment of these rhizobacterial isolates was found to activate the antioxidative defense mechanisms in rice seedlings through the induction of APX, SOD, chitinase, and PAL activities and accumulation of proline and polyphenols. This, therefore, indicated a significant role of the microbial inoculum in reducing the stress-induced ROS burden on host plants. We further evaluated the effect of six different rhizobacterial consortia, each of which is composed of five different rhizobacterial isolates on the stress tolerance of rice towards *R. solani* AG1-IA infection. Results showed a positive effect on disease tolerance by each of the six consortia formulations, although to different extents. To summarize, tea rhizobacteria have the potential to promote plant growth and modulating plant antioxidative defense systems, thereby aiding in biotic stress management in model plant systems. However, further studies are necessary to evaluate the usefulness of these PGP rhizobacteria in tea plantation. Finally, it is intriguing to explore the genome of individual rhizobacteria to find out their physiological robustness to become useful in the field conditions. Also, a study of the molecular cross-talk between the individual isolates of the different consortia is necessary to account for the relative efficiencies of the individual consortia in their plant growth promotion abilities.

## Supplementary information

Supplementary file1.
