# Supplementary material for: Evaluation of plant growth promotion properties and induction of antioxidative defense mechanism by tea rhizobacteria of Darjeeling, India
Source: Sci Rep. 2020 Sep 23;10:15536. doi: 10.1038/s41598-020-72439-z (PMC7511344; doi:10.1038/s41598-020-72439-z)

Supplementary Files

# Evaluation of plant growth promotion properties and induction of antioxidative defense mechanism by tea rhizobacteria of Darjeeling, India

Chandrima Bhattacharyya[1], Srimoyee Banerjee[2], Udita Acharya[2], Aroni Mitra[2], Ivy Mallick[1], Anwesha Halder[3], Shyamalina Haldar[4], Anupama Ghosh[2,]*, Abhrajyoti Ghosh[1,]*

[1]Department of Biochemistry, Bose Institute, Centenary Campus, P1/12, C.I.T. Road, Scheme VIIM, Kolkata 700054, India

[2]Division of Plant Biology[2], Bose Institute, Centenary Campus, P1/12, C.I.T. Road, Scheme VIIM, Kolkata 700054, India

[3]Department of Geography, University of Calcutta, 35 Ballygunge Circular Road, Kolkata-700019, India

[4]Department of Biochemistry, Asutosh College, University of Calcutta, Kolkata 700026, India

Supplementary Tables:

Table S1: Rhizobacterial consortium composition using a randomized design

Supplementary Figures:

Figure S1. Evaluation of plant-growth promoting traits upon individual treatments of selected tea rhizobacterial isolates on the Early Golden Bantam variety of maize seedlings. Seven days old seedlings were treated with individual rhizobacterial isolates, and the growth parameters such as (a) wet weight, (b) dry weight, (c) root length, and (d) shoot length were measured 15 days post-treatment. A two-sided t-test determined the significance level, and data are mean ± SD. (a: P-value- 0.05–0.01, b: P-value 0.01–0.001, and c: P-value less than 0.001)

Figure S2. Evaluation of Chlorophyll concentration upon individual treatments of selected tea rhizobacterial isolates on the Early Golden Bantam variety of maize seedlings. Seven days old seedlings were treated with individual rhizobacterial isolates, and the total chlorophyll concentration was measured 15 days post-treatment. A two-sided t-test determined the significance level, and data are mean ± SD. (a: P-value- 0.05–0.01, b: P-value 0.01–0.001, and c: P-value less than 0.001)

Figure S3. Effect of rhizobacterial treatment on (a) ascorbate peroxidase (APX), (b) catalase (CAT), (c) chitinase, and (d) phenylalanine ammonia-lyase (PAL) activity in root fraction of IR64 variety of rice seedlings. Five days old rice seedlings were treated with individual rhizobacterial isolates, and the antioxidative defense enzymes were measured in the root lysate preparation 14 days post-treatment. A two-sided t-test determined the significance level, and data are mean ± SD. (a: P-value- 0.05–0.01, b: P-value 0.01–0.001, and c: P-value less than 0.001)

Supplementary Files

Table S1. Rhizobacterial consortium composition using a randomized design

| Consortium | Rhizobacterial isolates |
|---|---|
| Group I | *Arthrobacter* sp. AB200, *Staphylococcus pasteuri* AB212, *Bacillus* sp. AB233, *Bacillus altitudinis* AB242, *Pseudomonas stutzeri* AB266 |
| Group II | *Exiguobacterium mexicanum* AB201, *Bacillus nakamurai* AB214, *Bacillus cereus* AB236, *Bacillus wiedmannii* AB246, *Ochrobactrum haematophilum* AB286 |
| Group III | *Leifsonia lichenia* AB203, *Bacillus atrophaeus* AB228, *Bacillus flexus* AB255, *Staphylococcus cohnii* AB312, *Ochrobactrum anthropi* AB285 |
| Group IV | *Bacillus niacini* AB209, *Bacillus velezensis* AB230, *Bacillus velezensis* AB237, *Bacillus megaterium* AB320, *Micrococcus luteus* AB321 |
| Group V | *Bacillus subtilis* AB267, *Staphylococcus gallinarum* AB328, *Bacillus thuringiensis* AB341, *Staphylococcus haemolyticus* AB336, *Bacillus pumilus* AB276 |
| Group VI | *Bacillus paralicheniformis* AB330, *Staphylococcus edaphicus* AB331, *Ochrobactrum* sp. AB345, *Lysinibacillus fusiformis* AB332, *Bacillus nitratireducens* AB304 |

Supplementary Files

Figure S1. Evaluation of plant-growth promoting traits upon individual treatments of selected tea rhizobacterial isolates on the Early Golden Bantam variety of maize seedlings. Seven days old seedlings were treated with individual rhizobacterial isolates, and the growth parameters such as (a) wet weight, (b) dry weight, (c) root length, and (d) shoot length were measured 15 days post-treatment. A two-sided t-test determined the significance level, and data are mean ± SD. (a: P-value- 0.05–0.01, b: P-value 0.01–0.001, and c: P-value less than 0.001)

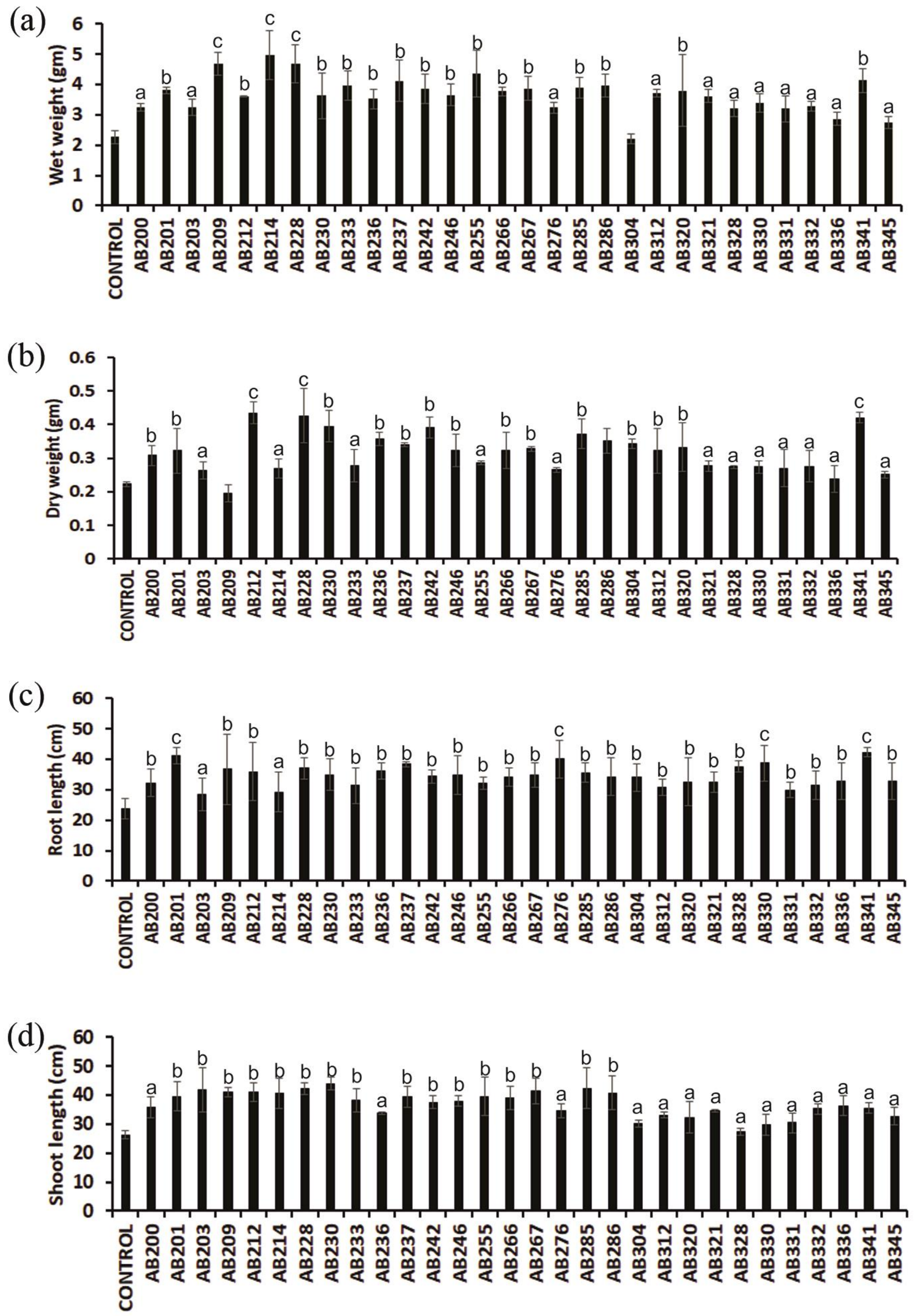

Figure S2. Evaluation of Chlorophyll concentration upon individual treatments of selected tea rhizobacterial isolates on the Early Golden Bantam variety of maize seedlings. Seven days old seedlings were treated with individual rhizobacterial isolates, and the total chlorophyll concentration was measured 15 days post-treatment. A two-sided t-test determined the significance level, and data are mean ± SD. (a: P-value- 0.05–0.01, b: P-value 0.01–0.001, and c: P-value less than 0.001)

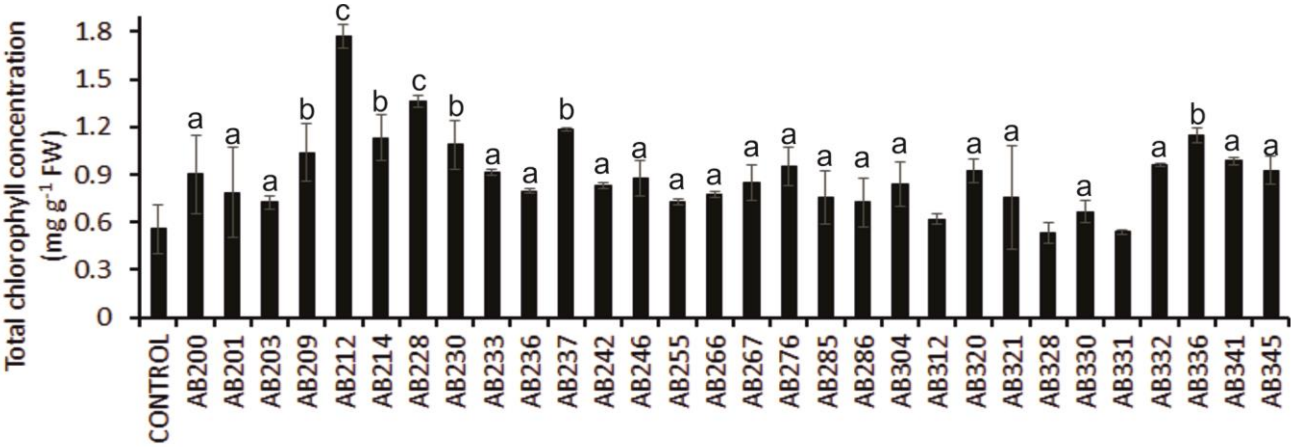

Figure S3. Effect of rhizobacterial treatment on (a) ascorbate peroxidase (APX), (b) catalase (CAT), (c) chitinase, and (d) phenylalanine ammonia-lyase (PAL) activity in root fraction of IR64 variety of rice seedlings. Five days old rice seedlings were treated with individual rhizobacterial isolates, and the antioxidative defense enzymes were measured in the root lysate preparation 14 days post-treatment. A two-sided t-test determined the significance level, and data are mean ± SD. (a: P-value- 0.05–0.01, b: P-value 0.01–0.001, and c: P-value less than 0.001)

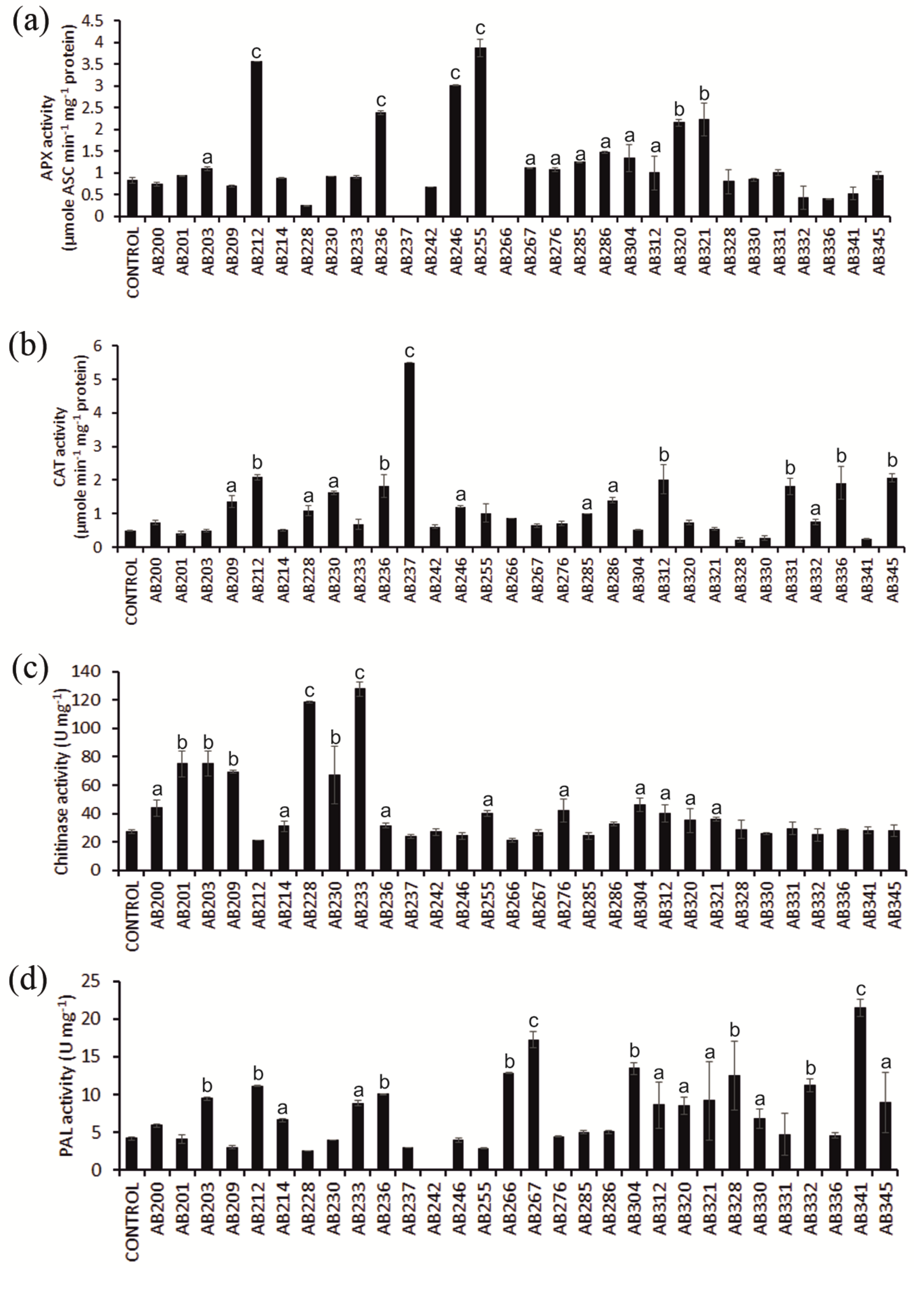

Supplement: Supplementary file 1 — Supplementary file1. [file 41598_2020_72439_MOESM1_ESM.pdf]
